# Long-Term Feeding of a High-Fat Diet Ameliorated Age-Related Phenotypes in SAMP8 Mice

**DOI:** 10.3390/nu12051416

**Published:** 2020-05-14

**Authors:** Hideaki Oike, Yukino Ogawa, Kayo Azami

**Affiliations:** Food Research Institute, National Agriculture and Food Research Organization (NARO), Kannondai 2-1-12, Tsukuba, Ibaraki 305-8642, Japan; yukino.ogawa@affrc.go.jp (Y.O.); azamik488@affrc.go.jp (K.A.)

**Keywords:** high-fat diet, senescence, lipid metabolism, circadian rhythm, chrono-nutrition

## Abstract

High-fat diets (HFD) have been thought to increase the risk of obesity and metabolic syndrome, as well as shorten lifespan. On the other hand, chrono-nutritional studies have shown that time-restricted feeding during active phase significantly suppresses the induction of HFD-induced obesity in mouse model. However, the long-term effects of time-restricted HFD feeding on aging are unknown. Therefore, in this study, we set up a total of four groups: mutual combination of ad libitum feeding or night-time-restricted feeding (NtRF) and an HFD or a control diet. We examined their long-term effects in a senescence-accelerated mouse strain, SAMP8, for over a year. Hearing ability, cognitive function, and other behavioral and physiological indexes were evaluated during the study. Unexpectedly, SAMP8 mice did not show early onset of death caused by the prolonged HFD intake, and both HFD and NtRF retarded age-related hearing loss (AHL). NtRF improved grip strength and cognitive memory scores, while HFD weakly suppressed age-related worsening of the appearance scores associated with the eyes. Notably, the HFD also retarded the progression of AHL in both DBA/2J and C57BL/6J mice. These results suggest that HFD prevents aging unless metabolic disorders occur and that HFD and NtRF are independently effective in retarding aging; thus, the combination of HFD and chrono-nutritional feeding may be an effective anti-aging strategy.

## 1. Introduction

High-fat diets (HFD), especially those rich in animal fat, are believed to promote aging, due to their undesirable impact on energy metabolism. In fact, laboratory animals fed such a diet in the typical diet-induced obesity (DIO) model develop various metabolic disorders, including abdominal obesity, hyperglycemia, hypertriglyceridemia, nonalcoholic fatty liver disease (NAFLD), and type 2 diabetes, leading to metabolic syndrome [1,2,3]. Recent chrono-nutritional studies have discovered that HFD attenuate the amplitude of the day and night eating rhythm, thereby attenuating the circadian rhythm of energy metabolism in various tissues and disrupting the energy balance of the whole body [4,5,6]. Therefore, when time-restricted feeding is performed during the active phase, the circadian rhythm amplitude is restored and the induction of obesity is significantly suppressed, even though the HFD contain the same calories as the ad libitum feeding [5,6]. However, it is not clear whether time-restricted feeding of HFD for the long term can prevent metabolic disorders.

Time-restricted feeding of normal diets improves energy metabolism in humans in short-term studies [7,8], even though calories are not restricted. Furthermore, long-term studies in experimental animal models showed that time-restricted feeding leads to cancer suppression and extended life span [9,10,11,12]. In other words, time-restricted feeding is a dietary method that promotes healthy longevity.

The Senescence-Accelerated Mouse (SAM) is a naturally occurring experimental mouse strain, which displays early aging phenotypes such as senile amyloidosis, senile osteoporosis, cataracts, impaired immune response, and deficits in learning and memory [13,14]. Several senescence prone (SAMP) lines have been established along with several senescence resistant (SAMR) lines. SAMP8 is a widely used model, especially in research on dementia, due to the early onset of brain atrophy, accompanied by learning/memory impairment and emotional disorders [15]. The lifespan of SAMP8 is almost half of SAMR1 [14,16].

Auditory aging, also known as age-related hearing loss (AHL), is mainly caused by an age-dependent decrease in the number of hair cells and auditory neurons in the inner ear [17]. Since these cells hardly regenerate, AHL is progressive and useful as a quantitative indicator of aging. In addition, caloric restriction, which is the most reliable anti-aging method beyond species, has been reported to delay the progression of AHL in experimental mice [17]. Similarly, the intake of some antioxidants, such as coenzyme Q10 and α-lipoic acid, also retards AHL progression [18,19]. It has also been reported that HFD retards AHL progression in relatively young C57BL/6 mice [20], suggesting that HFD possess anti-aging effects, unless metabolic disorders appear. In other words, it is possible that HFD delay aging if the metabolic disorders are prevented by the chrono-nutritional method. However, the long-term effects of time-restricted HFD feeding on aging and metabolism are unclear. Most studies using HFD have focused on metabolic abnormalities, and only few have exclusively assessed their effects on aging.

Here, we demonstrate the long-term effects of HFD on aging without remarkable metabolic disorders, using SAMP8 mice and the chrono-nutritional feeding method, night-time-restricted feeding (NtRF). We observed that some typical aging indicators, such as AHL, cognitive function, and appearance score, were improved by NtRF of the HFD.

## 2. Materials and Methods 

### 2.1. Animals

Animals were handled according to the guidelines of the Japanese Ministry of Agriculture, Forestry and Fisheries for laboratory animal studies, and the studies were reviewed and approved by the Animal Care and Use Committee of the Food Research Institute, NARO (approval number: H29-008, H29-039, and 19C004FRI).

Female SAMP8 mice (3 weeks old) and male DBA/2J mice (4 weeks old) were obtained from an institute for animal reproduction (Japan SLC, Hamamatsu, Japan). Male C57BL/6J mice (5 weeks old) were obtained from another institution (Charles River Japan, Yokohama, Japan). All mice were housed at 25 ± 1 °C, 50 ± 5% humidity, and a 12 h light-dark photocycle (ZT0 as light onset and ZT12 as light offset) and had ad libitum access to water. Body weight and water and diet consumption (per cage) were measured once a week during the experimental period.

### 2.2. Experimental Design and Time Course

**Figure 1:**
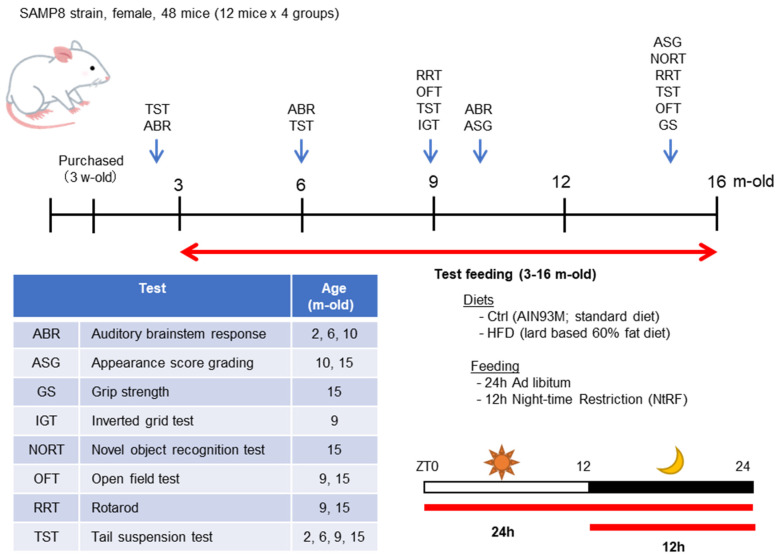
Time course of the experiment using SAMP8 mice: The female SAMP8 mice were divided into four groups (*N* = 12 each) and bred until 16 months old, using a combination of two diets and two feeding methods. The feeding period and test timing is indicated by red or blue arrows, respectively, within the timeline. The schematic diagram of the two feeding patterns are shown in the lower right. The contents and timing of the evaluation tests performed are described in the table.

The time course of the experiment using SAMP8 mice is described in Figure 1. The mice were divided into four groups (*N* = 12 each) when they were 5 weeks old and fed the test diet from 3 months old. During the experimental period, mice were fed either a standard diet (AIN93M [21]; Protein:Fat:Carbohydrate = 14.1:10.0:75.9, Oriental Yeast Co., Ltd., Tokyo, Japan) or a high-fat diet (HFD-60 [2], Protein:Fat:Carbohydrate = 18.2:62.2:19.6, Oriental Yeast). Each diet was fed ad libitum or 12 h night-time-restricted feeding (NtRF). Composition of the diets is shown in Table 1.

Behavioral tests and aging evaluation were performed at 2, 6, 9–10, and 15 months of age, as shown in Figure 1. Hearing was measured by auditory brain-stem response (ABR) at 2, 6, and 10 months of age. At 2 months old, only four mice from each group were randomly selected and tested, since all groups were under the same conditions. All the mice were tested for ABR at the ages of 6 and 10 months. A tail suspension test (TST) was performed as an index of depression [22], an open field test (OFT) was used as an index of anxiety-like behavior [23], an inverted grid test (IGT) was used as an index of motor strength/coordination [24], and a rotarod test (RRT) was used as an index of motor coordination [25]. The mice that survived at the age of 16 months (*N* = 6–7 each group) were anesthetized with an excess amount of sodium pentobarbital after a 4 h fast, blood samples were collected from the abdominal vena cava via laparotomy, and the mice were exsanguinated. Brain, liver, and femur were immediately dissected. The serum was isolated by centrifugation. The serum and liver samples were stored at −80 °C until subsequent analyses were performed. The femurs were immersed into 70% ethanol and stored at 4 °C until subsequent analyses were performed.

DBA/2J mice were provided the test diet from 5 weeks to 12 weeks old. Half of them were fed the standard diet (AIN93M), and the other half were fed the HFD (HFD-60) ad libitum (*N* = 8 each). At 5 and 12 weeks old, ABR were measured. 

C57BL/6J mice were provided the test diet from 3 to 9 months of age. Half of them were fed on the standard diet (AIN93M) and the other half were fed on the HFD (HFD-60) ad libitum (*N* = 8 each). At 3, 6, and 9 months old, ABR were measured.

### 2.3. Hearing Measurement

ABR were measured using TDT System 3 equipped with BioSigRP (Tucker Davis Technologies, Alachua, FL, USA), as described previously [26]. Mice were intraperitoneally anesthetized with a mixture of medetomidine (0.3 mg/kg), midazolam (4.0 mg/kg) and butorphanol (5.0 mg/kg) [27], and the needle electrodes were placed subcutaneously at the vertex and beneath the pinna of both ears. After the ABR measurement, atipamezole (0.3 mg/kg) was injected intraperitoneally to awaken the mice from anesthesia. The sound stimulus consisted of a 5 ms tone burst with a rise-fall time of 1.5 ms at frequencies of 8, 16, 24, and 32 kHz. The responses to 500 sweeps were averaged at each intensity level (5 dB steps) to assess the threshold. Hearing threshold was defined as the lowest stimulus intensity that produced reliable peaks in ABR waveforms. The better score was adopted as the threshold among the right and left ears. 

### 2.4. Other Aging Evaluation Tests

Novel Object Recognition Test (NORT): at 15 months of age, NORT was performed as an index of short memory. The NORT method was based on the one described previously [28], but with a few modifications. In brief, the test was performed between ZT12-16 in a dark cabinet equipped with an infrared camera. The mice were allowed to explore freely for 5 min in a cage with two fixed 50 mL plastic conical tubes (training session). After 10 min, one of the plastic tubes was randomly replaced with a conical flask, after which point the mouse was put back in the box and allowed to explore for 5 min (test session). We confirmed beforehand that there is no preference difference between both objects. The total time spent exploring was measured during training and test sessions. Exploration was defined as pointing the nose at the object at a distance of 2 cm or less and/or touching the object with the nose.

#### 2.4.1. Grip Strength

Muscle strength was evaluated by measuring grip strength at 15 months of age. The wire mesh was grasped by the forelimb of the mouse and pulled backward until it was released. The value of the spring scale linked to the wire mesh was recorded. The measurement was performed three times per mouse, and the best score was adopted.

#### 2.4.2. Grading Score

The grading score represents the senescent status according to the appearance of the SAM mouse [13,29]. Each item was graded with a score between 0 and 3, depending on the intensity of the change (grade 0 represented no particular change and grade 3 represented the most severe age-related change). Here, four items related to eyes were scored: periophthalmic lesions, corneal opacity, ulcer of the cornea, and cataract. The experimenter was blinded to grouping and other test results.

### 2.5. Blood Biochemical Analysis and Bone Density Measurement

Following biochemical indicators in serum were analyzed by Nagahama Life Science Laboratory (Nagahama, Japan) using routine laboratory methods; total protein (TP), albumin (ALB), albumin/globulin ratio (A/G), blood urea nitrogen (BUN), creatinine (CRE), aspartate aminotransferase (AST), alanine aminotransferase (ALT), alkaline phosphatase (ALP), lactate dehydrogenase (LDH), leucine aminopeptidase (LAP), amylase (AMY), creatine kinase (CK), total cholesterol (T-CHO), triglycerides (TG), glucose (GLU), and glycoalbumin (GA).

The bone density of the left femurs was measured by the single energy X-ray absorptiometry method at Kureha Special Laboratory (Tokyo, Japan). 

### 2.6. Gene-Expression Analysis

Total RNA was prepared from the frozen liver tissues using TRIzol RNA isolation reagent (Thermo Fisher Scientific, Waltham, MA, USA), according to the manufacture’s protocol, and cDNA was synthesized using ReverTra Ace reverse transcriptase (Toyobo, Osaka, Japan) with random hexamers. The transcripts were quantified using QuantStudio 3 realtime PCR system (Thermo Fisher Scientific) with Power SYBR Green Master Mix (Thermo Fisher Scientific) and the gene-specific primers (Supplementary Table S1). The relative amount of each transcript was normalized to the amount of Gapdh transcript in the same cDNA.

### 2.7. Statistical Analysis

All statistical analyses were carried out using EZR 1.41 based on R [30] or SIMCA 13.0.3.0. (Umetrics, Umeå, Sweden). The test methods applied, and the significant differences were described in each figure. Data were expressed as mean values ± standard error (SE). Heatmap was made using Microsoft Excel [31].

## 3. Results

### 3.1. HFD Did Not Induce Metabolic Disorders in SAMP8 Mice

**Figure 2:**
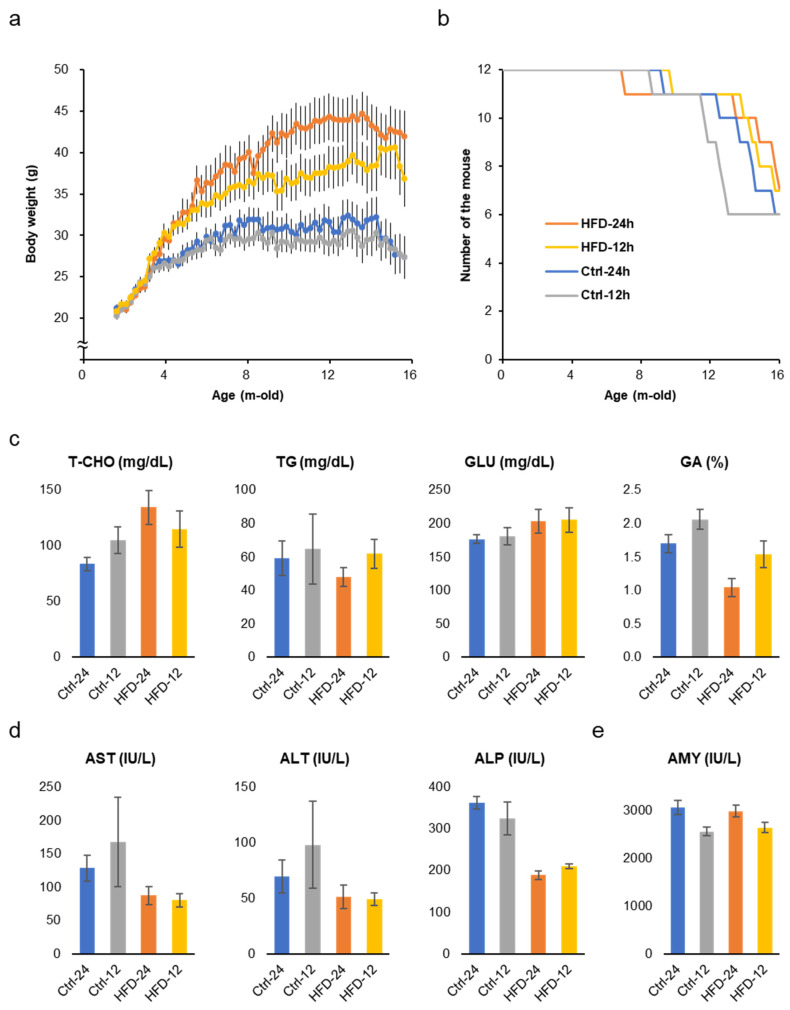
Body weight change, survival, and biomarkers in the serum: Changes in body weight (**a**) and survival (**b**) of SAMP8 mice throughout the experiment. Serum metabolic markers (**c**) and liver function markers (**d**) and a pancreatic marker (**e**) in 16 m-old SAMP8 mice (*N* = 6–7 each group). The results of all the measured biomarkers and statistics are shown in Supplementary Table S2. T-CHO: total cholesterol; GLU: glucose; TG: triglyceride; GA: glycoalbumin; AST: aspartate aminotransferase; ALT: alanine aminotransferase; ALP: alkaline phosphatase; AMY: amylase.

The two groups fed the HFD ad libtum and NtRF gained 30–50% more weight than the two groups fed the control diet (Figure 2a). NtRF suppressed the body weight by about 15% in the HFD group and about 5% in the control diet group. Weight gain suppression by NtRF was not as complete as in previous studies [5,6], while HFD overeating was suppressed by NtRF (Supplementary Figure S1a,b). Age-related changes in body weight peaked around the 8 month mark in the control diet groups, and were almost constant until around 14 months of age. On the other hand, it continued to increase in the HFD groups to around 14 months of age.

Early death due to metabolic disorders caused by HFD did not occur even in the ad libitum feeding group. Survival curves are statistically comparable for all four groups (Figure 2b and Supplementary Figure S1c,d, logrank test), though the group with lower average body weight tended to show earlier death onset. At 16 months of age, total cholesterol (T-CHO) level in serum was significantly high in the HFD groups, however, glucose (GLU) and triglyceride (TG) levels were comparable (Figure 2c and Supplementary Table S2). On the other hand, glycoalbumin (GA), that is, an index of the prevailing blood glucose concentrations over the preceding 2–3 weeks, was significantly higher in the control diet groups than in the HFD groups. AST and ALP, liver function markers, were also worse in the control diet groups (Figure 2d). Amylase (AMY), a marker of pancreatic function, showed better scores in the NtRF groups (Figure 2e). Bone density and brain weight at 16 months of age did not differ between the groups (Supplementary Figure S1e,f).

### 3.2. HFD Retarded AHL in SAMP8, DBA/2J, and C57BL/6J Mice

**Figure 3:**
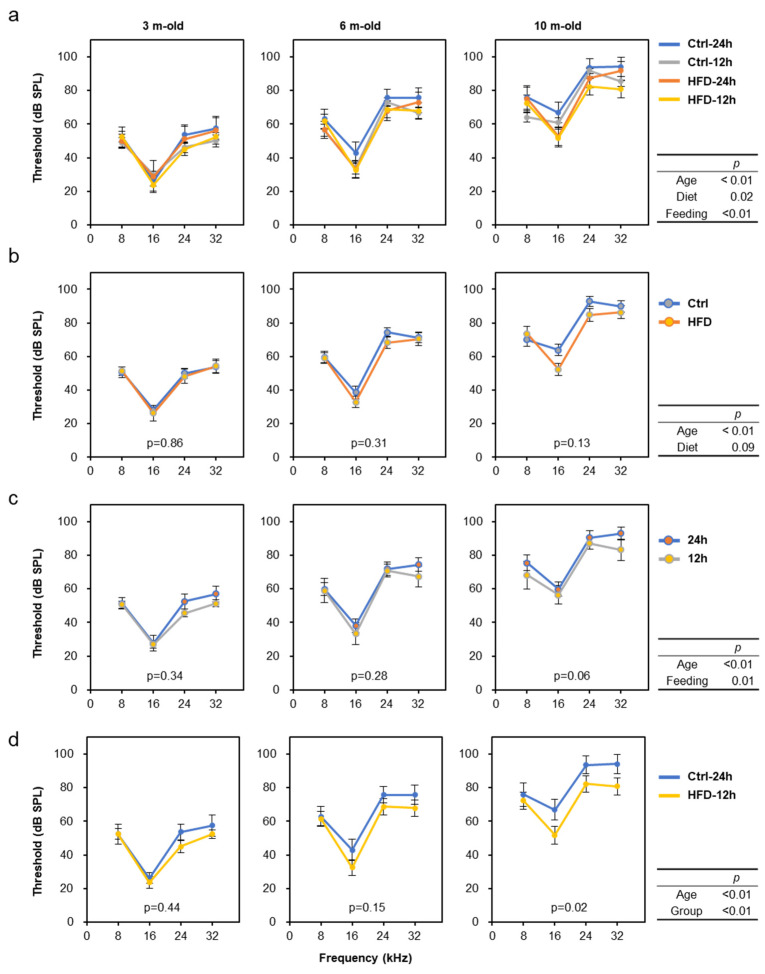
Age-related hearing decline and the nutritional effects in SAMP8 mice: Auditory brainstem response (ABR) were measured about 8, 16, 24, and 32 kHz at the age of 2, 6, and 10 m-old (randomly selected 4 mice each at 2 m-old and all 11–12 mice each at 6 and 10 m-old). Hearing threshold was defined as the lowest stimulus intensity in 5 dB steps. Data is shown by group (**a**), diet (**b**), feeding (**c**), and comparison of the two groups with the greatest difference in hearing at 10 months of age (**d**; Ctrl-24 h vs. HFD-12 h). The statistical *p*-values of multi-way repeated measures ANOVA are shown in the right table of each row, and the *p*-values by comparing one condition within each graph by one-way repeated measures ANOVA are shown in the figures.

Hearing tests were performed three times (at 2, 6, and 10 months of age) throughout the experimental period to evaluate the progression of age-related hearing loss (AHL) in SAMP8 mice. The results show that aging, diet, and feeding all affected the progression of AHL (Figure 3a; Multiway ANOVA; Age: *p* < 0.01, Diet: *p* = 0.02, Feeding: *p* < 0.01). For simplicity, when comparing the control diet and the HFD alone regardless of feeding time, we found that HFD intake mildly suppressed AHL progression (Figure 3b). Similarly, a comparison of free-feeding and time-restricted feeding without discriminating between diets revealed that NtRF suppressed AHL progression (Figure 3c). Compared to the ad libitum feeding of the control diet (the group with the worst hearing), the NtRF of the HFD (the best hearing group) significantly slowed the rate of auditory aging by approximately 20% (Figure 3d).

**Figure 4:**
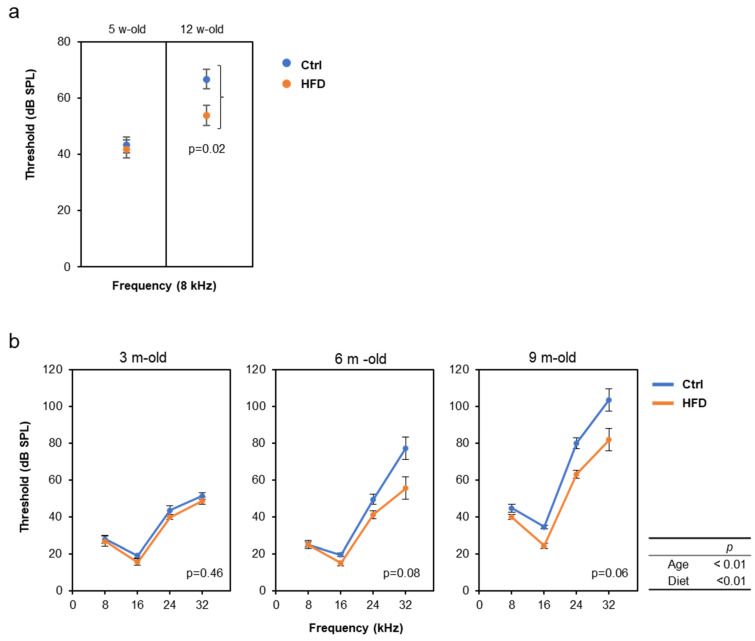
High-fat diet (HFD) attenuated age-related hearing loss (AHL) in DBA/2J and C57BL/6J mice: ABR were measured at the age of 5 and 12 w-old in male DBA/2J mice (**a**, *N* = 8 each) and at 3 and 9 m-old in male C57BL/6J mice (**b**, *N* = 8 each). Since the threshold at 12 w-old was above the measurement limit for frequencies above16 kHz, only 8 kHz results are used for DBA mice. The statistical *p*-values of two-way repeated measures ANOVA are shown in the right table in b, the *p*-values by comparing the diet condition with the t-test (**a**), or one-way repeated measures ANOVA (**b**) are shown in the figures.

We also examined the effects of the HFD on the progression of AHL using other two strains, DBA/2J and C57BL/6J, because it is known that the progression of AHL varies, depending on the mouse strains [32]. Especially, DBA/2J mice show rapid progression of AHL at the age of 2–3 months. Interestingly, the similar AHL prevention effect by the HFD was confirmed in both DBA/2J and C57BL/6J mice, despite the different time scales (Figure 4).

### 3.3. Effects of HFD and NtRF on Other Age-Related Phenotypes in SAMP8 Mice

The anti-aging effects of the HFD and NtRF on non-auditory age-related phenotypes were also evaluated in SAMP8 mice. Immobility time during tail suspension test (TST) is used as a depression index known to change with age [33]. We performed the TST at 2, 6, 9, and 15 months of age. Immobility time increased with age, but a significant difference was not detected among diet or feeding (Supplementary Figure S2a, two-way repeated measures ANOVA, *p* > 0.05). Similarly, the open field test (OFT), which is widely used as evaluation of anxiety, was performed at the age of 9 and 15 months, but no significant difference was observed between the groups (Supplementary Figure S2b). When the rotarod test (RRT) was performed at 9 and 15 months of age, as an index of physical ability, the average score tended to decrease with age, but was not significant between aging (Supplementary Figure S2c). Inverted grid test (IGT) was performed at 9 months of age to evaluate motor strength and coordination. The two HFD groups significantly registered a lower score than the control diet groups (Supplementary Figure S2d). However, there were obvious inverse correlations between individual body weights and scores for RRT and IGT, reflecting the difference in body weight rather than age-dependent motor ability.

**Figure 5:**
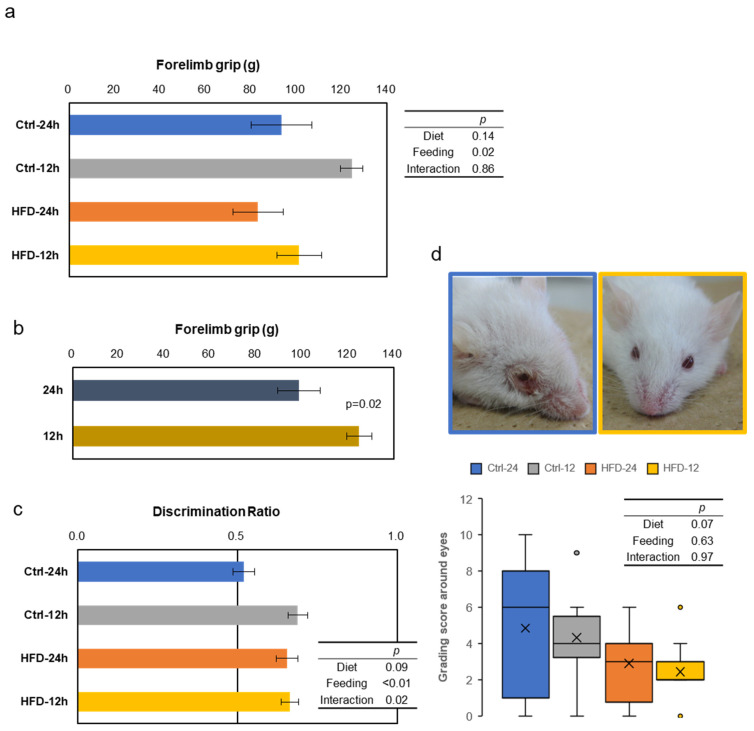
Other aging evaluation tests in SAMP8 mice: Behavioral tests were performed to compare effects of the diet and feeding on physical and mental performance with age. Results of grip strength (**a** and **b**), short memory by Novel Object Recognition Test (NORT) (**c**), and grading score about eyes with representative photos of the mice (**d**) at 15 m-old are shown (*N* = 6–7 in each group). The mouse on the left panel shows 10 points from the Ctrl-24 h group, and the mouse on the right panel shows 0 points from the HFD-12 h group. The statistical *p*-values of two-way ANOVA are shown in the right table (**a**,**c** and **d**), and the *p*-values by comparing one condition with t-test is shown in the figure (b, *N* = 12 each).

Grip strength test was performed at the age of 15 months, as the index of muscle strength. The scores were good in the NtRF groups, with significant effects regardless of the diets (Figure 5a,b). In the novel object recognition test (NORT), also conducted at the age of 15 months, the scores tended to be better, due to the HFD groups (Figure 5c, two-way ANOVA, *p* = 0.09 in diet). The effect of NtRF was significant (*p* < 0.01), and the score of the free-feeding group with the control diet was the worst. Furthermore, we noticed that the mice had less inflammation around the eyes in the HFD groups (Figure 5d). The tendency was confirmed at 15 m-old by the grading score, which is systematized from visual observation for SAM mice [29]. All four indicators showed similar trends, but the score of the corneal ulcer appeared to be the most strongly affected by diet.

### 3.4. HFD and NtRF Affected Hepatic Gene Expressions

**Figure 6:**
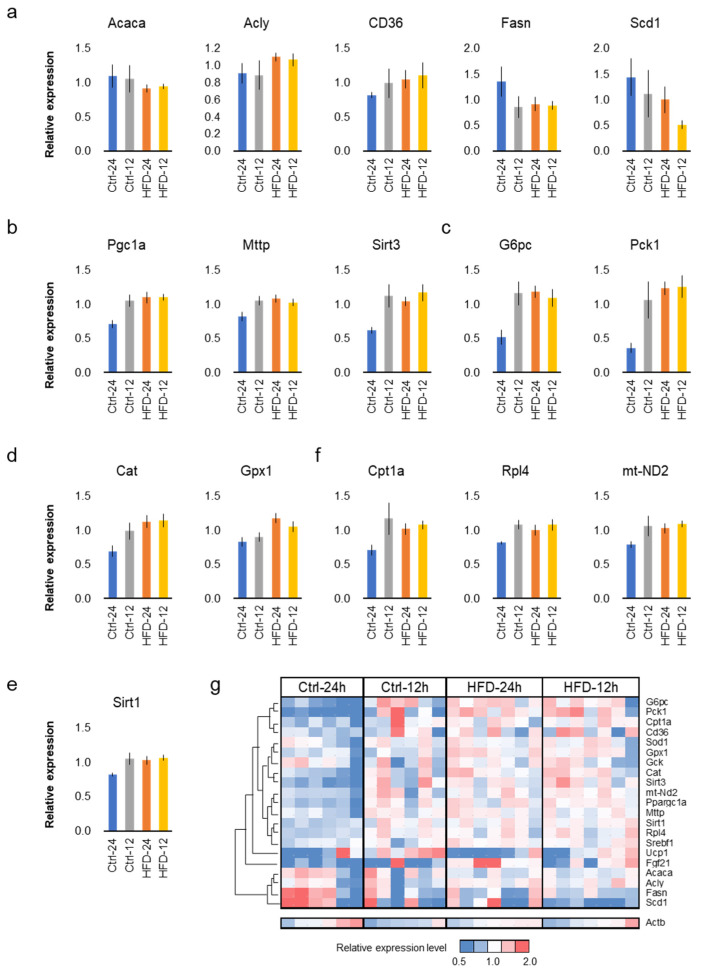
Hepatic gene expressions in SAMP8: hepatic gene expressions at 16 m-old associated with lipid metabolism (**a**), mitochondrial function (**b**), glucose metabolism (**c**), antioxidant (**d**), and anti-stress (**e**). Genes upregulated only by night-time-restricted feeding (NtRF) (**f**). Expression levels of all the 22 genes analyzed were visualized by heatmap with hierarchical clustering (**g**). The results of all the measured genes and statistics are shown in Supplementary Table S1.

Free feeding of the HFD did not seem to promote aging in SAMP8 mice. In order to support this at the molecular level, the comparative analysis of gene expression in the liver was performed among the four groups. Quantitative PCR (qPCR) was performed on 22 representative genes related to lipid metabolism (Figure 6a), mitochondrial function (Figure 6b), glucose metabolism (Figure 6c), and antioxidant/anti-stress (Figure 6d,e), as used in previous studies [34,35,36]. The results indicate that the diet and the feeding did not affect expression levels of representative genes in lipid metabolism, *Acaca*, *Acly*, *CD36*, *Fasn* and *Scd1* (Figure 6a and Supplementary Table S1, *p* > 0.05, two-way ANOVA, *N* = 6–7 each group). On the other hand, the free feeding group of the control diet tended to present low values for genes related to mitochondrial function, *Pgc1a* (*Ppargc1a*), *Sirt3*, *mt-ND2*, *Mttp*, and *Cpt1* (Figure 6b,f). This tendency was consistent in the glucose metabolic genes, *G6pc* and *Pck1* (Figure 6c) and antioxidant or anti-stress genes, *Cat*, *Sod1*, *Gpx1*, and *Sirt1* (Figure 6d,e and Table S1). NtRF upregulated *Cpt1a*, *Rpl4*, and *mt-ND2*, while the HFD did not affect them (Figure 6f and Table S1).

Heat map analysis of hepatic gene expression clearly showed that the expression pattern in the free-fed group of the control diet is significantly different from the other three groups (Figure 6g). The same tendency was also confirmed by cluster analysis and principal component analysis (Supplementary Figure S3).

## 4. Discussion

In this study, we showed that the lard based HFD delayed the onset of some aging phenotypes in mice. HFD have been considered to be unhealthy as they lead to metabolic syndrome; however, the SAMP8 mice were unexpectedly resistant to the undesirable impacts of the HFD, and did not cause early death due to metabolic disorders, which elicited the anti-aging effects of the HFD. In fact, the mice that were fed the HFD ad libitum gained 50% more body weight than those that were fed the control diet, but did not present a shortened lifespan as seen in other diet-induced obesity (DIO) model mouse [37]. In support of this, there were no metabolic abnormalities in blood glucose levels, serum biomarkers, and hepatic gene expressions, in spite of the 13 month-long consumption of the HFD in SAMP8. Thus, SAMP8 mice were characterized by a lower risk of metabolic disorders such as fatty liver, diabetes, and metabolic syndrome compared to other DIO model mouse. SAMP8 mice have the potential to provide novel insights in DIO studies. Interestingly, a previous study showed that SAMP8 mice fed with n-6 fatty acids rich safflower oil lived longer than those fed n-3 fatty acids rich perilla oil [38]. The nutritional studies using mouse models suggest that the lipid metabolism of SAMP8 mice is different from that of DIO mouse strains such as C57BL/6, because the lipid-induced longevity is generally caused by the n-3 fatty acids, rather than the n-6 fatty acids [39]. This might be the reason why metabolic disorders did not occur in SAMP8 mice, even when the HFD was fed for almost entire life spans. Though the anti-aging effects of the HFD in SAMP8 mice may need to be carefully interpreted in view of such specific metabolism, HFD retarded AHL also in DBA/2J and C57BL/6J mice, indicating that at least some of the anti-aging effects of HFD are not due to strain-specific phenotypes. On the one hand, lard might be the main factor in the anti-aging effect of HFD. In a long-term calorie restriction experiment using C57BL/6 mice, it was reported that lard as a fat source prevented aging of the skeletal muscle, compared to soybean or fish oil [40]. The ultrastructure and function of mitochondria was improved by lard, which might be close to the present study. On the other hand, high fat itself might be the main factor. A recent study showed that long-term feeding of a ketogenic diet, which is composed of high fat (mainly vegetable oil) and low carbohydrate, to C57BL/6 mice alternated weekly reduced midlife mortality and improved memory in old age [41]. This feeding method did not induce obesity, indicating that both concept and results are consistent with our study. Since normal DIO model mice cannot be used in the long-term demonstration because of the shortened lifespan by metabolic disorders, it is considered that the anti-aging effects of HFD have not been reported.

Hepatic gene expression analysis showed that the Ctrl-24h group differed significantly from the other three groups (Figure 6g). The expression pattern of typical genes, commonly used in energy metabolism and anti-aging studies, showed the clear effects of HFD and NtRF, which are consistent with the results of blood biomarkers and behavioral tests. HFD and NtRF contributed to the increased expression of genes associated with mitochondrial and antioxidant functions. It might be possible that the consumption of HFD and NtRF reduced tissue damage by reactive oxygen species (ROS), mainly produced in mitochondria, because mitochondrial ROS are the primary cause of aging in both mouse and human hearing systems [42,43,44,45,46]. For example, the reduction of ROS through genetic modification (MCAT mouse, that over-express catalase in mitochondria), caloric restriction, or antioxidant intake slows the progression of AHL in mice [17,18]. In this study, we could not provide direct evidence that suppression of ROS production by HFD or NtRF led to an anti-aging phenotype. To clarify the mechanism, time series analysis is effective in the next step. At that time, the AHL-delaying effect of HFD, which takes several months in SAMP8 mice, can be reproduced in several weeks in DBA/2J mice, which should be useful as an anti-aging research model.

Previous findings suggest that NtRF of HFD could reduce body weight gain to levels close to free feeding of normal diets [5,6], but its efficacy in this study was modest. However, this does not indicate that time-restricted feeding was ineffective. Since HFD-induced overeating was suppressed (Figure S1a,b), it is considered that HFD-induced eating dysregulation was at least partially eliminated. Past studies have been biased towards C57BL/6 mice. It is possible that the effects of time-restricted feeding on body weight are different between strains, because the lipid metabolism is different, as mentioned above. The effects of time-restricted feeding are known to improve energy metabolism and suppress cancer growth, even though caloric intake is not reduced [7,11]. In addition, disorder of the circadian rhythm promotes aging by disturbing the immune system [47]; conversely, it is expected that aging may be delayed by increasing the amplitude of the circadian rhythm via regular NtRF. Consistent with the anti-aging effects shown in a number of studies, our results suggest that NtRF improved hearing, learning/memory, and muscle strength scores in old mice. In addition, consumption of the HFD significantly suppressed AHL and moderately improved memory function and appearance score. The anti-aging effects of the HFD interacted partially with the effects of the NtRF, but were independent for the most part, suggesting that their action pathway differs. Indeed, hearing, the most quantitative age-related indicator, showed an additive effect of HFD and NtRF. This indicates that the quality and extent of the effect can be increased by combining HFD and NtRF.

**Figure 7:**
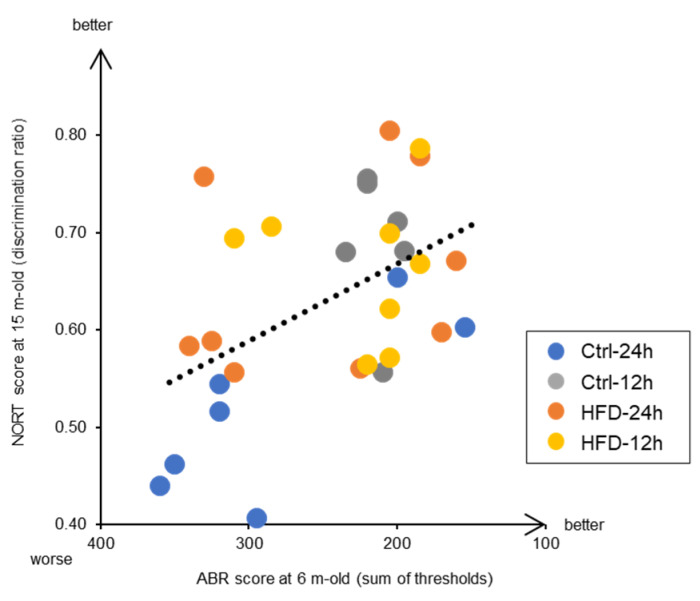
Correlation between middle-aged hearing and old-aged memory in SAMP8 mice: Individual results of 15 m-old NORT (Figure 5) and 6 m-old ABR (Figure 3) are plotted, and the correlation was statistically tested based on Peason’s product-moment correlation coefficient (*R* = −0.49, *p* < 0.01, *N* = 30).

Moreover, this study also reveals interesting findings related to the prevention of aging of brain functions, which happens in dementia. We found a significant correlation between the hearing ability at the early middle age of 6 months and the memory test score at the old age of 15 months (Figure 7). This indicates that individuals with early auditory aging also have early cognitive deficits. Both AHL and brain aging are promoted by neurodegeneration. In other words, both inner ear and brain neurodegeneration have the common underlying mechanism, where neurons are damaged by ROS and either die or stop functioning [48]. Indeed, catalase overexpression in mitochondria is effective not only in delaying AHL [18], but also preventing dementia in Alzheimer’s disease model mice by neuroprotection [49]. Recent epidemiological studies in humans have shown that hearing loss increases the risk of dementia, and that hearing loss prevention is most likely to contribute to dementia prevention [50,51,52,53]. Since the mouse model used here was confirmed to resemble the phenotype of human aging, at least in the context of correlation between AHL and dementia, it would be useful in future research on the prevention of neuronal aging. In addition, it is expected that the potential application of HFD and NtRF in the development of anti-aging foods and dietary methods in the future.

## 5. Conclusions

Long-term feeding of the HFD over most of the lifespan in SAMP8 mice revealed anti-aging effects of the diet when it did not cause metabolic disorders. In addition, combining HFD with time-restricted feeding elicited greater anti-aging effects.

## Figures and Tables

**Table 1:** Composition of experimental diets.

Ingredient (g/100 g Diet)	AIN93M (Control Diet)	HFD-60 (High-Fat Diet)
Casein	14.0	25.6
L-Cystine	0.18	0.36
Corn starch	46.6	-
α-Corn starch	15.5	16.0
Sucrose	10.0	5.5
Soybean oil	4.0	2.0
Lard	-	33.0
Maltodextrin	-	6.0
Cellulose	5.0	6.61
AIN93G mineral mix	3.5	3.5
AIN93 vitamin mix	1.0	1.0
Calcium carbonate	-	0.18
Choline bitartrate	0.25	0.25
Tert-butylhydroquinone	0.0008	-
Total	100.0	100.0
Calorie (kcal/100 g diet)	360	493

## Supplementary Materials

The following are available online at https://www.mdpi.com/2072-6643/12/5/1416/s1, Figure S1: Cumulative food intake, brain weight, survival curves, bone density, and heatmap of blood biomarkers, Figure S2: Results of tail suspension test, open field test, rotarod test, and inverted grid test, Figure S3: Hierarchical cluster analysis and PCA analysis about hepatic gene expressions, Table S1: Information and results of quantitative PCR. Table S2: Results of blood biochemistry.
