# Supplementary material for: Long-Term Feeding of a High-Fat Diet Ameliorated Age-Related Phenotypes in SAMP8 Mice
Source: Nutrients. 2020 May 14;12(5):1416. doi: 10.3390/nu12051416 (PMC7285040; doi:10.3390/nu12051416)
Supplement: Supplementary file 1 [file nutrients-12-01416-s001.zip › Supplementary files 200501/Figure S1 200428.pdf]

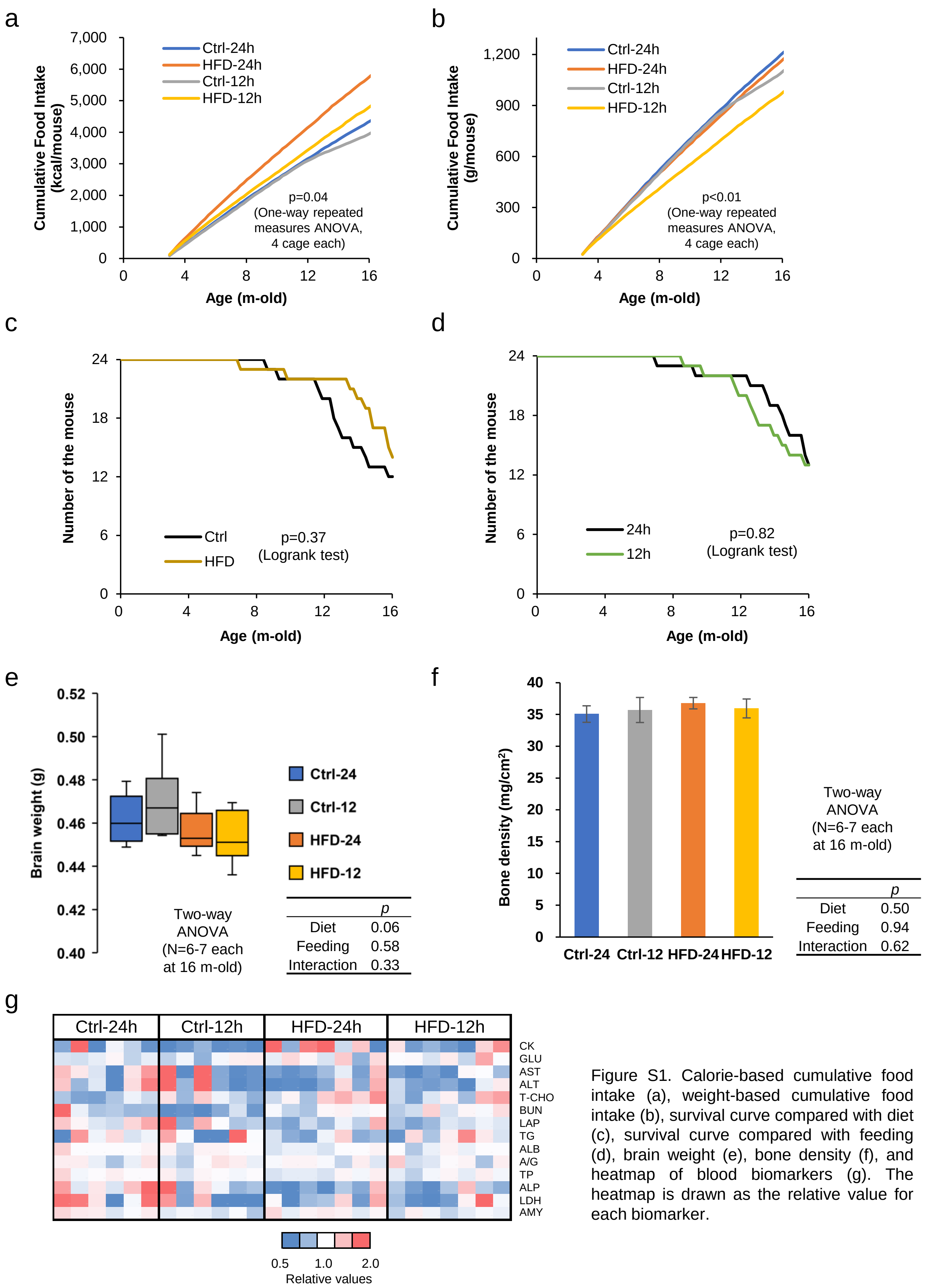

Figure S1. Calorie-based cumulative food intake (a), weight-based cumulative food intake (b), survival curve compared with diet (c), survival curve compared with feeding (d), brain weight (e), bone density (f), and heatmap of blood biomarkers (g). The heatmap is drawn as the relative value for each biomarker.
