# Supplementary material for: Long-Term Feeding of a High-Fat Diet Ameliorated Age-Related Phenotypes in SAMP8 Mice
Source: Nutrients. 2020 May 14;12(5):1416. doi: 10.3390/nu12051416 (PMC7285040; doi:10.3390/nu12051416)
Supplement: Supplementary file 1 [file nutrients-12-01416-s001.zip › Supplementary files 200501/Figure S2 200428.pdf]

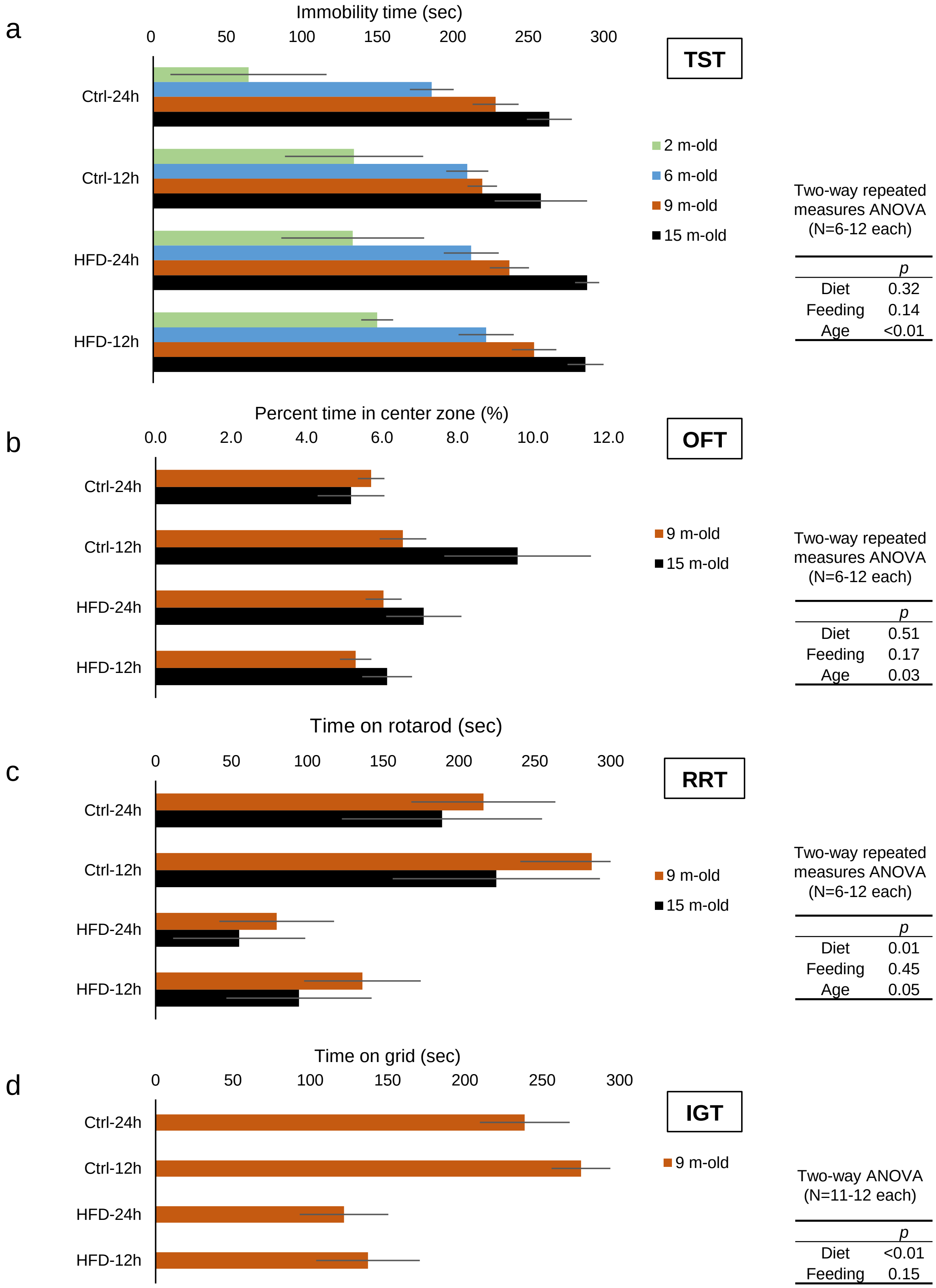

Figure S2. Results of tail suspension test (TST), open field test (OFT), rotarod test (RRT), and inverted grid test (IGT)
