# Supplementary material for: Long-Term Feeding of a High-Fat Diet Ameliorated Age-Related Phenotypes in SAMP8 Mice
Source: Nutrients. 2020 May 14;12(5):1416. doi: 10.3390/nu12051416 (PMC7285040; doi:10.3390/nu12051416)
Supplement: Supplementary file 1 [file nutrients-12-01416-s001.zip › Supplementary files 200501/Figure S3 200430.pdf]

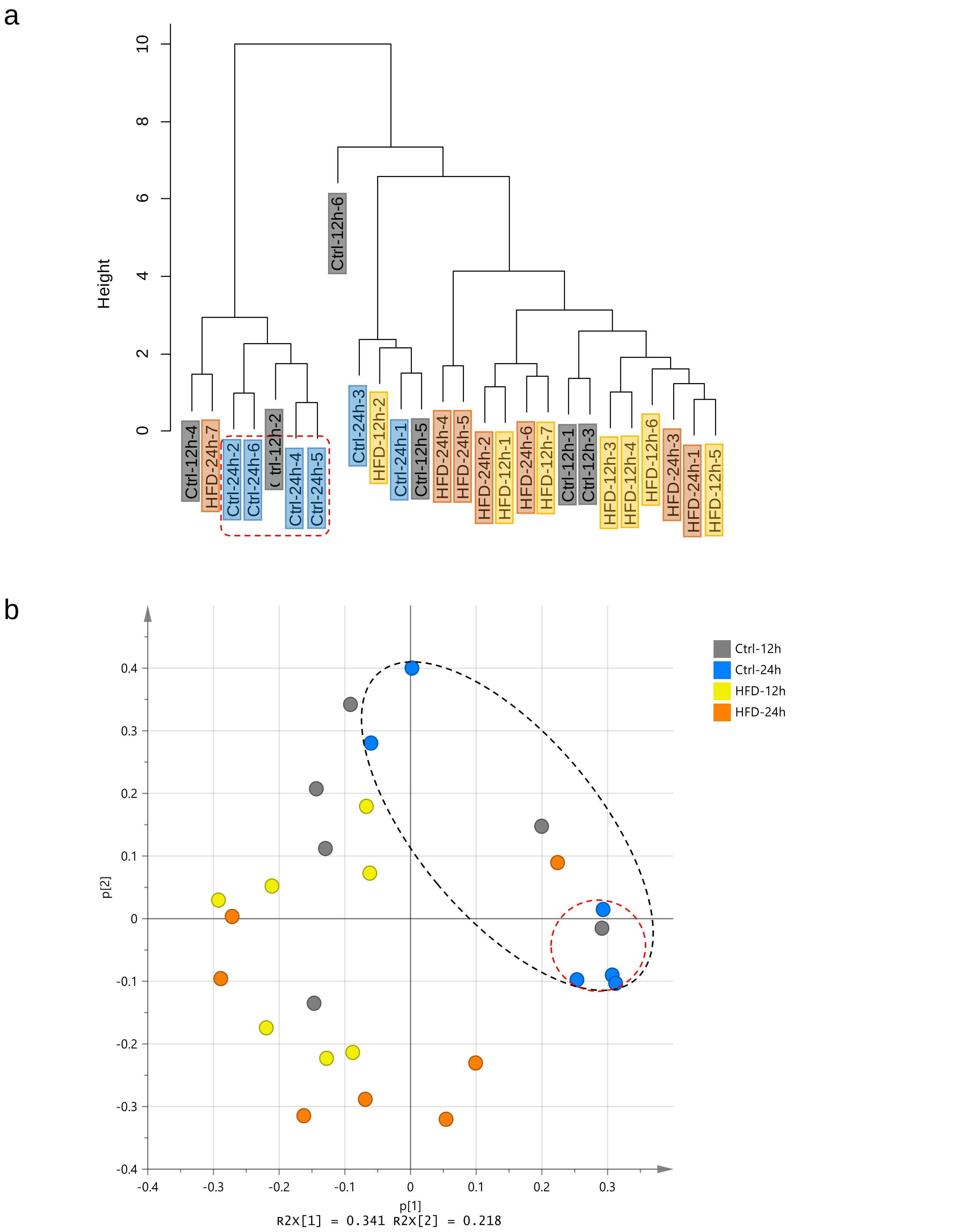

Figure S3. Hierarchical cluster analysis (a) and PCA analysis (b) about hepatic gene expressions
Four out of six mice or all six mice in Ctrl-24h group were assigned to the same cluster (a) or close distance (b), respectively.
